# Target organ expression and biomarker characterization of chemokine CCL21 in systemic sclerosis associated pulmonary arterial hypertension

**DOI:** 10.3389/fimmu.2022.991743

**Published:** 2022-09-23

**Authors:** Henriette Didriksen, Øyvind Molberg, Adi Mehta, Suzana Jordan, Vyacheslav Palchevskiy, Håvard Fretheim, Einar Gude, Thor Ueland, Cathrine Brunborg, Torhild Garen, Øyvind Midtvedt, Arne K. Andreassen, Fridtjof Lund-Johansen, Oliver Distler, John Belperio, Anna-Maria Hoffmann-Vold

**Affiliations:** ^1^ Department of Rheumatology, Oslo University Hospital, Rikshospitalet, Oslo, Norway; ^2^ Department of Immunology, University of Oslo and Oslo University Hospital, Oslo, Norway; ^3^ Department of Rheumatology, University Hospital Zurich, University of Zurich, Zurich, Switzerland; ^4^ Department of Medicine, David Geffen School of Medicine at University of California, Los Angeles (UCLA), Los Angeles, CA, United States; ^5^ Department of Cardiology, Oslo University Hospital, Rikshospitalet, Oslo, Norway; ^6^ Research Institute of Internal Medicine, Oslo University Hospital – Rikshospitalet, Oslo, Norway; ^7^ Oslo Centre for Biostatistics and Epidemiology, Research Support Services, Oslo University Hospital - Rikshospitalet, Oslo, Norway

**Keywords:** systemic sclerosis, CCL21, pulmonary arteria hypertension, ELISA Enzyme-linked immunosorbent assay, Luminex (xMAP) method, Anti-CCL21

## Abstract

**Introduction:**

Systemic sclerosis (SSc) is a heterogenous disorder that appears to result from interplay between vascular pathologies, tissue fibrosis and immune processes, with evidence for deregulation of chemokines, which normally control immune trafficking. We recently identified altered levels of chemokine CCL21 in SSc associated pulmonary arterial hypertension (PAH). Here, we aimed to define target organ expression and biomarker characteristics of CCL21.

**Materials and methods:**

To investigate target organ expression of CCL21, we performed immunohistochemistry (IHC) on explanted lung tissues from SSc-PAH patients. We assessed serum levels of CCL21 by ELISA and Luminex in two well-characterized SSc cohorts from Oslo (OUH, n=552) and Zurich (n=93) University hospitals and in 168 healthy controls. For detection of anti-CCl21 antibodies, we performed protein array analysis applying serum samples from SSc patients (n=300) and healthy controls. To characterize circulating CCL21 in SSc, we applied immunoprecipitation (IP) with antibodies detecting both full length and tailless and a custom-made antibody detecting only the C-terminal of CCL21. IP products were analyzed by SDS-PAGE/western blot and Mass spectrometry (MS).

**Results:**

By IHC, we found that CCL21 was mainly expressed in the airway epithelial cells of SSc patients with PAH. In the analysis of serum levels of CCL21 we found weak correlation between Luminex and ELISA (r=0.515, p<0.001). Serum levels of anti-CCL21 antibodies were higher in SSc patients than in healthy controls (p<0.001), but only 5% of the SSc population were positive for anti-CCL21 antibodies in SSc, and we found no correlation between anti-CCl21 and serum levels of CCL21. By MS, we only identified peptides located within amino acid (aa) 23-102 of CCL21, indicating that CCL21 in SSc circulate as a truncated protein without the C-terminal tail.

**Conclusion:**

This study demonstrates expression of CCL21 in epithelial lung tissue from SSc patients with PAH, and indicate that CCL21 in SSc circulates as a truncated protein. We extend previous observations indicating biomarker potential of CCL21, but find that Luminex is not suitable as platform for biomarker analyses. Finally, *in vivo* generated anti-CCL21 antibodies exist in SSc, but do not appear to modify serum CCL21 levels in patients with SSc-PAH.

## Introduction

Systemic sclerosis (SSc) is a complex multi-organ disorder with highly heterogeneous phenotypes that appear to be shaped by complex interactions between immune-mediated inflammatory processes leading to distinct serum autoantibodies, uncontrolled fibrosis of skin and internal organs and vascular pathology leading to small vessel vasculopathy (1). Since SSc often runs a progressive course and responds poorly to standard-of-care therapies, it associates with high disease burden and reduced life expectancy (2). To improve management of SSc, we need new therapeutics, and we need to understand how to select the right patients for the right therapies at the right time; and how to evaluate the effects of the therapies (2, 3) These key questions are possible to address, but to do so, we first need better understanding of the disease evolution and outcome across all SSc phenotypes and the specific organ-systems targeted by the disease.

Other than skin biopsies, the lack of tissue samples as sources for biological assessments represents a major hurdle for organ specific analyses. Thus, circulating biomarkers have been extensively studied to identify parameters allowing patient stratification to predict overall and organ-specific disease evolution and outcome across SSc populations (4, 5). Although promising candidates are emerging, their development into clinical use is largely lacking (6). Standardization of methods has been a significant hurdle in this regard.

Given their critical role in immune cell function, it is hardly surprising that chemokines appear important for the development and propagation of autoimmune diseases; and may represent useful biomarkers for disease evolution. In SSc, it was early shown that the CC chemokine 2 (CCL2) was important for the development of tissue fibrosis (7). More recently, studies indicate biomarker potential of additional chemokines including CCL18, CX3CL1 and CXCL4 in SSc-associated interstitial lung disease (5, 8–10).

Pulmonary arterial hypertension (PAH) leading to right heart failure is an organ manifestation that develops in 10-15% of the SSc patients (11). Unfortunately, we have limited understanding of risk factors for the development of PAH in SSc (12, 13). Therefore, we currently screen all SSc patients regularly for PAH with strategies that are time and cost effective to capture cases at early stages (14). This makes PAH a prime example of the unmet need to identify specific markers of organ manifestation to tailor and individualize patient management. In 2018, we reported chemokine CCL21 as a promising serum marker for the development of PAH in SSc patients. In that study, we assessed levels of CCL21 in serum samples by a commercial enzyme linked immunosorbent analysis (ELISA) with a commercially available anti-CCL21 monoclonal antibody to capture soluble ligands (15). This CCL21 ELISA worked well for our proof-of-concept purpose, but like most other in-house ELISAs, it was time-consuming, required large serum volumes and was not very suitable for standardization. Hence, to come a step closer for inclusion of CCL21 as a parameter for personalized PAH approaches in SSc patients, there was a need for development of a more standardized CCL21 assay. Ideally, one would like to have a standard assay combining high sensitivity and high throughput, allowing rapid assessment of not only CCL21, but also other promising PAH markers, in small amounts of blood.

Originally, we proposed CCL21 as a circulating marker of immune-mediated inflammatory processes driving the small vessel obliteration that causes PAH in SSc. Support for this hypothesis came from murine studies linking receptor CCR7 and it cognate ligands CCL19 and CCL21 to lung inflammation and PAH development (16–18). Additionally, there were observations indicating deregulation of CCR7 and/or CCL21 in human PAH (19, 20).

Very recently, we have focused on potential links between lymph vessel modulating factors other than CCL21 and development of SSc-PAH. This research was based on studies demonstrating abnormal lymphatic vessels in SSc, and linked abnormal lymph vessels to human, genetically determined PAH variants (21). Since it appeared that CCL21 effects on lymph vessels were often mediated through vascular endothelial growth factor-C (VEGF-C), we focused particularly on this factor, and its cognate receptor VEGFR-3. We found associations between serum VEGF-C and PAH, and multivariable regression analysis showed that VEGF-C and soluble VEGFR-3 predicted PAH development (22).

In this current study, we return to CCL21, and its potential role as a marker of pathways important for SSc. Specifically, we approach four different, but linked theoretical and practical research questions that require attention.

The first question addresses the expression of CCL21 in the lung of SSc patients with PAH. Previously, in lung cancer, it has been shown that CCL21 is mainly expressed in lymphatic vessels (23). To date, there is limited knowledge on expression of molecular markers in lung tissue from patients with SSc-PAH. The major reason for this is that lung biopsies are not taken from patients with PAH due to high risk of procedure-related bleedings, limiting availability of tissue to lung explants and autopsy material. Hence, there are no studies on the expression pattern of CCL21 is expressed in lung tissue from SSc-PAH patients.

Second, we address the question on why some SSc-patients with PAH have low levels of circulating CCL21. Early *in vitro* experiments demonstrated that monoclonal antibodies generated against specific chemokines can block chemokine-receptor binding and inhibit receptor-mediated signaling pathways (24). Interestingly, some of the antibodies generated against chemokines appear to have major diagnostic and therapeutic purpose, as is the case for monoclonal antibodies against CCL21, which is applied for diagnostic and therapeutic purpose in inflammatory bowel disease (25) and to reduce fibrocytes in renal fibrosis (26). Recent evidence suggests that humans, under certain circumstances, generate functional antibodies targeting different chemokines and cytokines (27). This type of auto-antibodies appear to block signaling and drive disease phenotypes in subsets of patients with primary immune deficiencies, and they seems to be prevalent in some infectious diseases, including COVID-19, and in autoimmune diseases (28, 29). From these observations, we hypothesized that some patients might have low levels of circulating CCL21 because of the presence of specific anti-CCL21 antibodies.

Third, we address properties of circulating CCL21. As stated above, CCL21 and CCL19 are both ligands of the receptor CCR7. CCL21 is 32 amino acids longer than CCL19 on the C-terminal end, they share only 32% amino acid identity and have different signal effects through CCR7 (30, 31). Interestingly, protease activity in dendritic cells (DCs) forms a naturally occurring truncated product of CCL21, lacking the C-terminal end. It appears that this product is equally potent as CCL19 in inducing DC chemotaxis (32). It is not clear if circulating CCL21 in patients with SSc exists as a full-length or truncated protein, or a mixture of both. This question is potentially important for understanding signaling properties of CCL21 in SSc-PAH.

Last, but not least, we aimed to develop a new, standardized and more versatile assay for circulating CCL21. We focus on the Luminex X multi-analyte profiling (MAP) analysis. While ELISA measures singular ligands, Luminex xMAP analysis is a high throughput method able to measure several ligands at the same time making it less time-consuming and less expensive than ELISA. However, xMAP has a higher risk of cross-reactivity between antibodies than ELSIA, and the specificity and comparability between the two methods are important (33). Studies have investigated the comparability of ELISA and Luminex, finding good correlations between the methods, i.e in Alzheimer’s studies detecting cerebrospinal fluid biomarkers (34, 35).

Taken together, we aimed in this study to investigate properties of circulating CCL21, potential role of *in vivo* generated anti-CCL21 antibodies and expression of CCL21 in lung tissue from SSc patients developing PAH. Moreover, we aimed to assess the reproducibility, specificity and comparability between ELISA and Luminex xMAP in the measurement of CCL21 in serum samples from SSc patients.

## Methods

### Study cohort, PH screening and diagnostic procedure

The study cohort included 552 patients form the Oslo University Hospital (OUH) SSc cohort and 93 patients from the Zurich University Hospital (USZ) SSc cohort. We have previously shown that clinical and demographic data were similar in the SSc cohort at OUH and USZ (22), and the cohorts were therefore assembled for the serum analyses in this study. We also included tissue samples from four SSc patients from the University of California, Los Angeles (UCLA).

All included patients met the 2013 EULAR/ACR classification criteria for (36, 37). To enrich for patients with pulmonary hypertension (PH), we included all patients from the OUH and USZ cohorts who had conducted a right heart catheterization (RHC). Standard procedures for PH screening in the OUH and USZ SSc cohort include complete clinical examination, echocardiography, pulmonary function tests, 6-minute walk test, NT-proBNP and evaluation by the DETECT algorithm. Patients are referred to RHC if clinical suspicion of PH and/or the DETECT score >35 (38, 39). PH was diagnosed by RHC according to the 2015 ESC guidelines (40). SSc-PAH was defined by the presence of pre-capillary PH with a mean pulmonary artery pressure (mPAP) ≥25 mmHg and pulmonary capillary wedge pressure (PCWP) <15 mmHg. Unlike PH-ILD, the absence of significant lung fibrosis (<10% extent of lung fibrosis on HRCT at OUH; and <20% extent of lung fibrosis by visual scoring at USZ) are also a criterion for PAH diagnosis. Patients with PCWP ≥ 15 mmHg were diagnosed as post-capillary PH (38, 41, 42). We had two healthy controls populations, one who included 100 random serum samples from the blood bank at OUH, and a second with 68 additional healthy controls matching the SSc cohort by age and gender. For the anti-CCL21 analyses, we applied a total of 1192 serum samples from patients with connective tissue diseases (SSc (n=300), Sjogren’s syndrome (SS) (n=148), systemic lupus erythematosus (SLE) (n=345) and mixed connective tissue disease (MCTD) (n=239)), all fulfilling the respective classification criteria and diagnosis by rheumatology experts at OUH, and healthy donors (n=160). Of the 300 SSc patients included in this analysis, 216 (72%) SSc patients were from the study cohort described above.

### Immunohistochemistry

To assess the expression of CCL21 in lung tissue, we performed immunohistochemistry (IHC). Collection of lung biopsies for diagnostic or therapeutic purpose in patients with high pulmonary arterial pressure is not recommended, as detailed in the introduction. Therefore, the standard IHC staining was performed on paraffin-embedded samples of lung tissues from one SSc-PAH patient obtained at lung transplantation at OUH, two autopsy material from SSc-PAH and two SSc-ILD patients undergoing lung transplantation at UCLA. The Vectastain ABC Elite kits (Vector Laboratories, Inc., Burlingame, CA, USA) were used as described (43), with 1:500 dilution of 100µg/ml rat polyclonal anti-human CCL21 (GTX31167, GeneTex, Irvine, CA, USA). We also stained alveolar macrophages with 1:100 dilution of FLEX monoclonal mouse anti-human CD68, PG-M1 clone (GA613, Agilent). Stained slides were counterstained with hematoxylin, mounted and analyzed for cellular sources of specific chemokine. Negative controls were conducted and stained without primary antibody.

### CCL21 analysis in serum samples

Blood samples were collected at OUH and USZ. They were processed within 30 min after collection and stored at -70°C until analysis, following standardized procedures as described in the EUSTAR guidelines on biobanking (44). At OUH, blood samples are drawn at inclusion in the Norwegian Systemic Connective Tissue Disease and Vasculitis Registry (NOSVAR) (45). The serum samples were assessed by two different methods: ELISA and Lumiex xMAP.

### Enzyme linked immunosorbent assay

This assay has been described in detail (2018): Briefly, we applied a two-step binding process of primary antibody and labelled secondary antibody (indirect ELISA). Detection of CCL21 were achieved with the Human CCL21/6Ckine DuoSet ELISA from R&D systems with a 1:1 dilution of serum and assay buffer. A 30-40% solution of calf-serum were used to prevent non-specific binding to antigens and antibodies. The serum samples were analyzed in duplicates in a 96-well tray. A seven-point standard curve were created with a serial dilution of recombinant Human CCL21 standard, ranging from 62.5 pg/ml to 4000 pg/ml.

### Luminex xMAP analysis

For Luminex analysis the Milliplex^®^ Human Cytokine/Chemokine Panel II kit containing CCL21 was used. The serum samples (1:1:1 serum/buffer/serum matrix solution) and antibody immobilized beads (Anti-Human 6CKine beads) were loaded on 96-well plates in duplicates and run on Luminex^®^ MAGPLEX machine and software. A six-point standard curve were created using serial dilution solution of Human Cytokine/Chemokine Panel II standard, ranging from 19.5 to 20 000 pg/ml.

**Figure 1:**
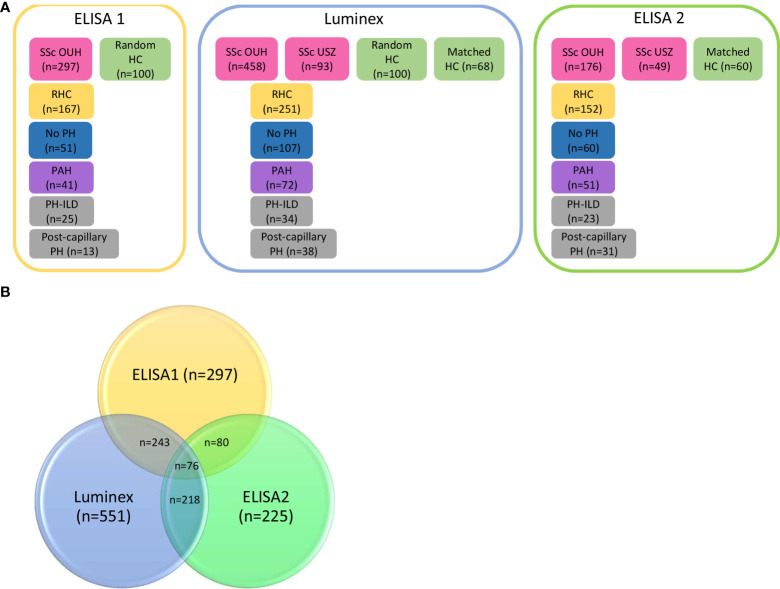
Schematic overview of the number of systemic sclerosis (SSc) patients included in ELISA 1, Luminex and ELISA 2. **(A)** In the previously published ELISA 1, 297 SSc patients and 100 random healthy controls were included. Of these 167 patients had undergone by RHC and 41 patients were diagnosed with PAH. In the Luminex analysis, serum samples from 458 SSc patients form Oslo University Hospital (OUH), 93 serum samples form SSc patients from Zurich University Hospital (USZ) and the random healthy controls were run, as well as 68 age and gender matched healthy controls. In total, 251/456 patients included in the Luminex analysis had undergone RHC and 72 were diagnosed with PAH. For the follow-up ELISA 2, 176 serum samples form OUS, 49 serum samples from USZ and 60 age and gender matched healthy controls were included. Of these patients, 152 had conducted RHC and 51 were diagnosed with PAH. **(B)** The samples overlapping in the three different runs were 243 samples between ELISA1 and Luminex, 80 in ELISA1 and ELISA2, and 218 in Luminex and ELISA 2. The number of samples overlapping in all three runs were 76 samples.

For these analyses, we used previously published results from the ELISA analysis as reference (15), For simplicity, we here refer to the published data as ELISA1. As shown in **Figure 1**, the ELISA1 was run with serum samples from 297 patients from the SSc cohort at OUH and 100 random healthy controls (15).

Luminex xMAP: For this analysis, we included serum samples from 458 patients from OUH, of which 197 of the serum samples were re-analyzed by ELISA 1, and 93 serum samples included from USZ. As controls, we included the same 100 random healthy controls from ELISA1 and 68 new controls matched for age and gender to the SSc population.

ELISA 2: To confirm and extend findings from the previous analyses, we set up a second ELISA including serum samples already analyzed by ELISA 1 (n=80) or Luminex xMAP (n=225). These included 176 SSc samples form OUH, 49 SSc samples from USZ, and 60 healthy controls gender and age matched to the SSc cohort.

### Assessment of anti-CCL21 antibodies in serum samples

This analysis was performed as an integral part of a custom human protein array aiming for broad detection of serum auto-antibody specificities. Microsphere beads were conjugated with GST-tagged Human CCL21 Recombinant Protein (H00006366-P01, Abnova) and serum IgG autoantibodies were detected with R-phycoerythrin-conjugated goat anti-human IgG antibody (Jackson Immunoresearch; cat. no. 109-005-008). Serum profiling was performed on an Attune NxT flow cytometer (ThermoFisher Scientific), equipped with 4 lasers: 405 nm (Pacific Blue, Pacific Orange), 488 nm (Alexa Fluor 488), 567 nm (R-phycoerythrin) and 633 nm (Alexa Fluor 647, Alexa Fluor 750). Data files exported from the flow cytometer were processed with a freely available R application designed for the analysis of thousands of parameters in each sample simultaneously, identifying all the microspheres subsets by means of the variable color codes retrieving the median R-phycoerythrin values for each subset. The dataset was subjected to median normalization across all samples (46).

### Immunoprecipitation, mass spectrometry and western blot

Since CCL21 is found both in full length and in a truncated version, we wanted to investigate which forms of CCL21 were present in serum samples from SSc patients using immunoprecipitation (IP), mass spectrometry (MS) and Western blot (WB). First, antibodies detecting CCL21 were tested against full length CCL21, and a product of CCL21 missing the C-terminal tail called “tailless CCL21” on an SDS-page gel followed by Western blot for their specificity. Full length CCL21 was purchased from R&D systems and tailless CCL21 (SDGGAQDCCL KYSQRKIPAK VVRSYRKQEP SLGCSIPAIL FLPRKRSQAE LCADPKELWV QQLMQHLDKT PSPQKPAQG) was produced by ALMAC (**Table 1**).

**Table 1:** Chemokines and antibodies used in SDS/PAGE and Western blot analysis.

Product	Supplier	Catalog number	Working concentration
**Recombinant Human CCL21/6Ckine**	R&D systems	366-6C-025	10µg/ml
**Tailless CCL21**	ALMAC	Custom-made	10µg/ml
**Anti-human CCL21/6Ckine antibody**	R&D systems	MAB366-1	1-2µg/ml
**Anti-human CCL21/6Ckine antibody**	R&D systems	MAB366	1-2µg/ml
**CCL21 Monoclonal antibody**	ThermoFisher	MA5-23751	1-2µg/ml
**Human CCL21/6Ckine antibody**	R&D systems	AB366	1µg/ml
**Anti-CCL21 polyclonal antibody**	Sicgen	Custom-made	1-5µg/ml

IP was performed using Dynabeads™ Protein G Immunoprecipitation Kit from ThermoFisher Scientific, following the kit user manual. We used 1000 µl serum samples from two SSc patients with high serum levels of CCL21 on ELISA and Luminex xMAP. CCL21 antibody detecting amino acid sequence in the middle of CCL21 was purchased from ThermoFisher Scientific (MA5-23751, 10 µg/ml) and anti-CCL21 polyclonal goat antibody detecting the C-terminal was purchased from Sicgene Antibodies (10 µg/ml). Proteomics were carried out by enzymatic digestion of the IP products by trypsin followed by mass spectrometry on a Q Exactive™ Hybrid Quadrupole-Orbitrap™ Mass Spectrometer connected to an EASY-nLC 1000 Liquid Chromatograph, both from ThermoFisher Scientific, identifying peptides my mass fingerprinting. The IP products were also loaded on an SDS-page gel (Any kD™ Mini-PROTEAN^®^ YGX™ PREcast Protein Gel) followed by Western blotting as a control for the mass spectrometry, using anit-CCL21 from ThermoFisher scientific (MA5-23751), and polyclonal donkey anti-mouse IgG from Jackson Immunoresearch (715-035-150). Precision Plus Protein™ Dual Xtra was used as standard for size interpretation and Thermo Scientific Pierce G2 Fast Blot machine was used for Western blotting. Detailed protocols are shown in supplementary material.

### Statistical analysis

Applications used for statistical analyses were IBM SPSS version 28 and STATA version 17. Pearson Chi-square test, Fischer exact test, or independent sample t-test were used as appropriate. For correlation of CCL21 levels using ELISA and Luminex, we applied Spearman’s rho; a correlation coefficient between 0.7 and 1 was considered as a strong correlation. We determined a cut-off value for “high” and “low” CCL21 serum levels by receiver operated curves (ROC), where area under the curve (AUC) values >0.7 were considered acceptable. To assess CCL21 properties regarding its association with PAH, we applied logistic regression analyses with odds ratio (OR) and 95% confidence interval (CI) to analyze risk factor associated with PAH. We included all PAH patients from the ELISA2 cohort with clinical data and serum samples collected from one year before to 5 years after PAH diagnosis. To analyze CCL21s predictive ability for PAH we applied Cox regression analysis with hazard ratio (HR) and 95% CI. Here, we included patients with PAH from the ELISA 2 cohort with clinical data and serum samples collected >6 months prior to PAH diagnosis. Variables for logistic- and cox-regression analysis were chosen based om previous results (15). For both analyses, patients from the ELISA 2 cohort diagnosed with no PH were added as control group. For the antibody analysis, the standard normal distribution table was used to calculate a Z-score determining which antibodies had a value within the 10% highest scores. Patients were considered positive for anti-CCL21 if they had a z-score > 1.28.

## Results

### Expression of CCL21 in lung tissue specimen from patients with SSc-PAH

**Figure 2:**
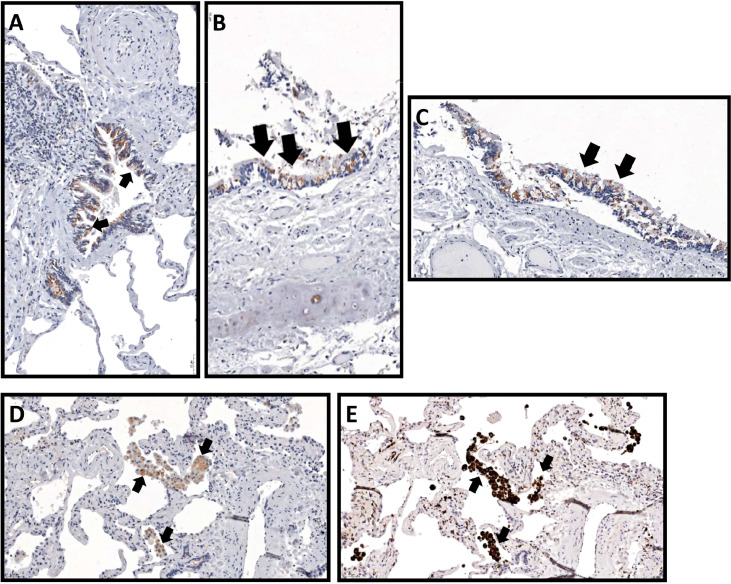
CCL21 expression in systemic sclerosis with associated pulmonary arterial hypertension from explanted lung tissue. CCL21 immunohistochemistry demonstrated protein expression (black arrows) from **(A-C)** airway epithelial cells (20x) and **(D)** alveolar macrophages (20x), which was confirmed by **(E)** CD68 macrophage marker in a serial cut of the same histopathology block.

We performed IHC in explanted lung tissue collected from three patients with SSc-PAH, and lung autopsies from two deceased SSc-PAH patients. In all these patients, lung tissues stained positive for CCL21. The staining showed predominant expression of CCL21 in airway epithelial cells (**Figures 2A-C**), but we were also able to detect expression of CCL21 in alveolar macrophages (**Figure 2D**). The expression of CCL21 in alveolar macrophages was confirmed by staining a serial cut of the same histopathology block with macrophage marker CD68 (**Figure 2E**).

### Analysis of CCL21 in serum samples from SSc patients

**Figure 3:**
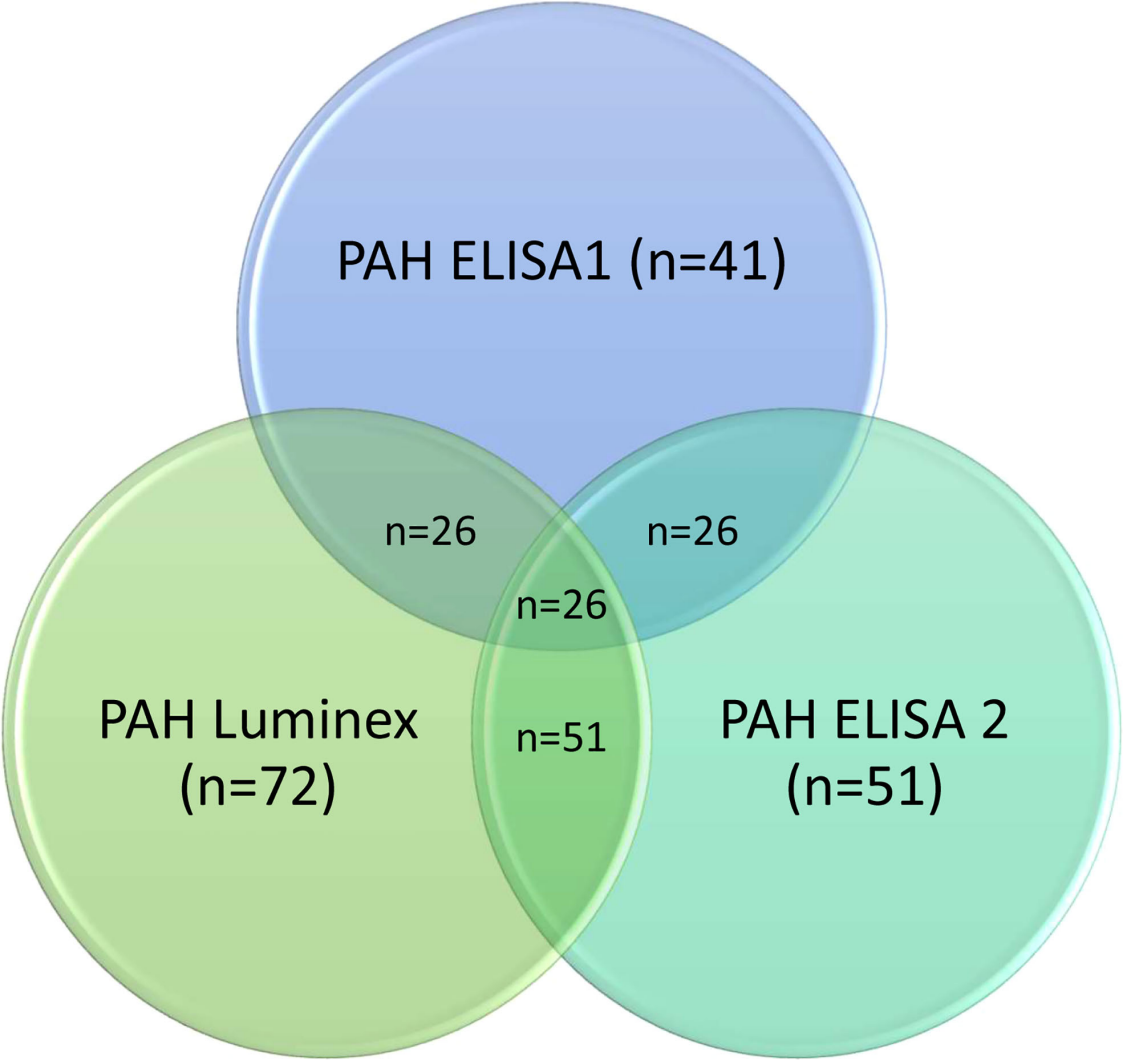
Overlapping PAH patients in ELISA1, Luminex and ELISA2. The number of PAH patients overlapping in ELISA1 and Luminex were 26 patients. Serum samples from these 26 patients were also included in ELISA. The number of patients overlapping in Luminex and ELISA2 were 51 patients.

To replicate and extend previous results by ELISA indicating serum CCL21 as a marker and predictor for PAH in SSc (15), we performed two independent rounds of experiments applying Luminex and ELISA for serum CCL21 quantification. To facilitate comparison between all the experiments, we included in this study some key results from our published primary ELISA work, designated ELISA1. Demographic and clinical characteristics of the previous ELISA1 (n=297) and the current Luminex (n=551) and ELISA2 (n=225) patient cohorts were comparable (**Table 2**). As shown in **Figure 1B**, we included a proportion of the patients from ELISA1 in Luminex and/or ELISA2. The frequency of SSc patients with PAH diagnosis verified by RHC were comparable between the three cohorts, being 24.3% (41/169) in ELISA1, 28.7% (72/251) in Luminex, and 22.7% (51/165) in ELISA 2. As shown in **Figure 3**, we were able to include 26 of the 41 SSc-PAH serum samples from ELISA1 in both Luminex and ELISA2 experiments.

**Table 2:** Key demographic and clinical data of SSc patients in the ELISA 1, Luminex and the ELISA 2.

	ELISA 1 (n = 297)	Luminex (n = 551)	ELISA 2 (n = 225)
**Age at disease onset, years**	48.7 (15.3)	51.4 (15.4)	52.2 (15.1)
**Males, no (%)**	55 (18.5)	93 (16.9)	50 (22.2)
**Diffuse cutaneous, no (%)**	78 (26.3)	119 (21.6)	51 (22.7)
**Anti-centromere Ab, no (%)**	113 (38.0)	284 (51.4)	111 (50.0)
**NYHA class, no (%)**	230 (57.9)	496 (89.9)	204 (90.7)
** NYHA class 1-2, no (%)**	171 (43.1)	403 (80.9)	151 (74.0)
** NYHA class 3-4, no (%)**	59 (14.8)	93 (18.7)	53 (26.0)
**NT-pro BNP, pg/mL**	73.8 (304.9)	86.5 (359.2)	95.2 (350.6)
**DLCO %**	68.2 (21.8)	68.1 (20.0)	65.1 (19.0)
**Examined by ECHO, no (%)**	291 (97.9)	457 (82.8)	198 (88.0)
** sPAP mmHg**	27.8 (19.2)	27.0 (17.4)	31.9 (21.4)
**Examined by RHC, no (%)**	169 (56.9)	251 (45.5)	165 (73.3)
** mPAP, mmHg**	28.9 (13.2)	25.6 (11.1)	28.8 (10.6)
** PAWP, mmHg**	9.6 (7.3)	9.5 (5.9)	10.2 (6.6)
** PVR, WU**	3.7 (4.0)	3.7 (6.4)	3.9 (7.6)
** PAH, no (%)**	41 (24.3)	72 (28.7)	51 (22.7)
**Time from disease onset to PH, years**	9.1 (8.8)	8.1 (9.9)	7.7 (9.9)
**Time from serum sampling to PH, years**	-0.33 (2.3)	-0.78 (3.1)	-0.84 (3.0)
**Therapy, no (%)**	123 (41.4)	284 (51.4)	152 (67.6)
**Immunosuppressive, no (%)**	50 (16.8)	141 (25.5)	76 (33.8)
**PAH-treatment**	87 (29.3)	156 (28.3)	92 (40.9)
** Mono therapy, no (%)**	42 (48.3)	76 (48.7)	43 (46.7)
** Duo therapy, no (%)**	40 (45.9)	67 (42.9)	40 (43.5)
** Triple therapy, no (%)**	5 (5.7)	13 (8.3)	9 (9.8)
**Calcium channel blockers, no (%)**	35 (11.8)	153 (27.7)	78 (34.7)
**ACE-inhibitors, no (%)**	13 (4.4)	56 (10.1)	33 (14.7)
**Deceased, no (%)**	87 (29.3)	126 (22.9)	64 (28.4)

The clinical and demographic data did not significantly differ in the OUH and USZ cohort, except from the percentage of deceased patients (22). Values are the mean (SD) if not indicated otherwise. NYHA, New York Heart Association; NT-pro BNP, N-terminal pro-Brain Natriuretic Peptide; DLCO, diffusing capacity of carbon monoxid; ECHO, Echocardiography; sPAP, systolic pulmonary artery pressure; RHC, right heart catheterization; PH, pulmonary hypertension; PAH, pulmonary arterial hypertension; mPAP, mean pulmonary artery pressure; PAWP, pulmonary artery wedge pressure; PVR, pulmonary vascular resistance; Mono therapy, phosphodiesterase-5 inhibitors (PD5I) or endothelin receptor antagonists (ERA); Duo therapy, PD5I and ERA; Triple therapy, PD5I, ERA and prostacyclins (P); ACE-inhibitors, Angiotensin converting enzyme-inhibitors.

### Serum levels of CCL21 in SSc-PAH

**Figure 4:**
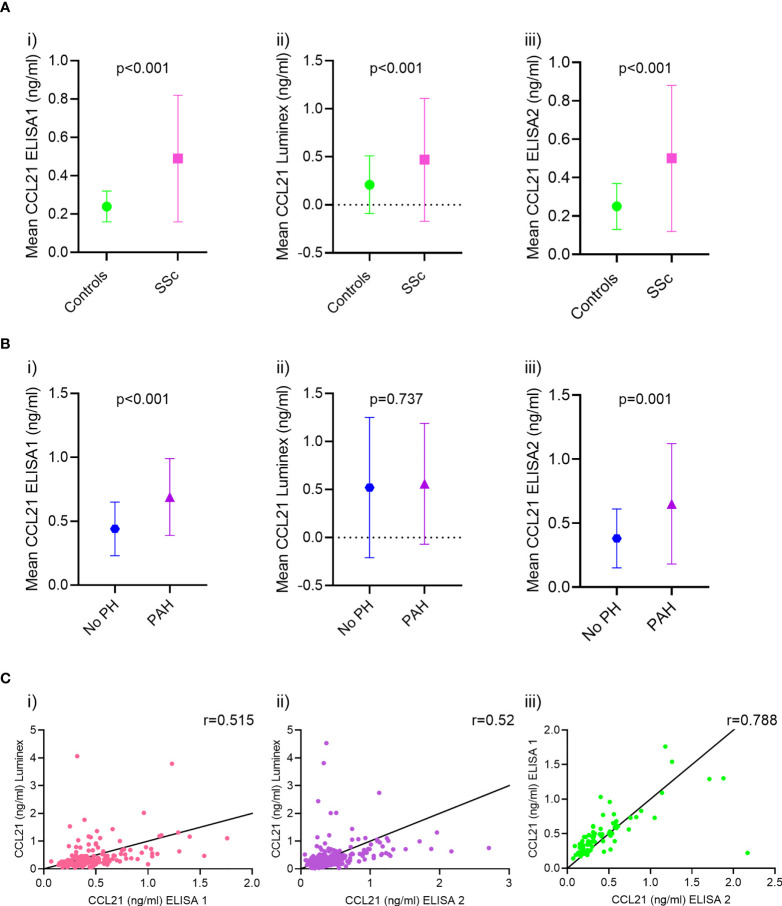
CCL21 serum levels and correlation analysis in ELISA 1, Luminex and ELISA 2. **(A)** The mean serum levels of CCL21 in ELISA 1 were higher in SSc patients compared to healthy controls (i) in Luminex (ii) and ELISA 2 (iii). **(B)** In ELISA 1, CCL21 serum levels were higher in patients diagnosed with PAH compared to patients with no PH (i) while similar in PAH patient and no PH (ii) in Luminex. The mean serum levels of CCL21 in ELISA 2 however were, like in ELISA 1, higher in PAH patients compared to those without PH (iii). **(C)** The correlation of CCL21 serum levels between ELISA 1 and Luminex (r=0.515, p<0.001) **(i)** and the correlation of CCL21 serum levels between ELISA 2 and Luminex (r=0.52, p<0.001) (ii) were poor. However, the correlation of CCL21 serum levels between ELISA 2 and ELISA 1 (r=0.788, p<0.001) (iii) were good.

In the previously published ELISA1 cohort (15), serum levels of CCL21 were higher in SSc patients than healthy controls (0.49 ± 0.33ng/ml vs. 0.24 ± 0.08ng/ml, p<0.001) (**Figure 4A**, i). CCL21 levels were also higher in SSc-PAH patients (n=41) compared to SSc patients with no PH (n=51) by RHC (0.69 ± 0.30ng/ml vs. 0.44 ± 0.21ng/ml, p<0.001) (**Figure 4B**, i).

In the Luminex xMAP analysis, the serum levels of CCL21 were higher in SSc patients than healthy controls (0.47 ± 0.81ng/ml vs. 0.21 ± 0.30ng/ml, p<0.001) (**Figure 4A**, ii), as we saw in ELISA1. However, in the Luminex analyses, CCL21 levels were similar in SSc-PAH patients (n=72) compared to SSc patients with no PH (n=107) by RHC (0.56 ± 0.63ng/ml vs. 0.52 ± 0.73ng/ml, p=0.737) (**Figure 4B**, ii).

In the current ELISA2, serum levels of CCL21 were higher in SSc patients than healthy controls (0.50 ± 0.38ng/ml vs. 0.25 ± 0.12ng/ml, p<0.001) (**Figure 4A**, iii). In ELISA2 CCL21 levels were higher in SSc-PAH patients (n=51) compared to SSc patients with no PH (n=60) by RHC (0.66 ± 0.46ng/ml vs. 0.38 ± 0.23 ng/ml, p=0.001) (**Figure 4B**, iii).

The serum levels of CCL21 obtained by the ELISA1, Luminex xMAP and ELISA 2 were correlated with each other. The correlation between ELISA1 and Luminex xMAP, and ELISA2 and Luminex xMAP were significant but only moderate, r=0.515 (p<0.001) and r=0.52 (p<0.001), respectively (**Figure 4C**, i-ii). However, the correlation between ELISA 1 and ELISA 2 were significantly stronger with a correlation coefficient of r=0.788 (p<0.001) (**Figure 4C**, iii).

**Figure 5:**
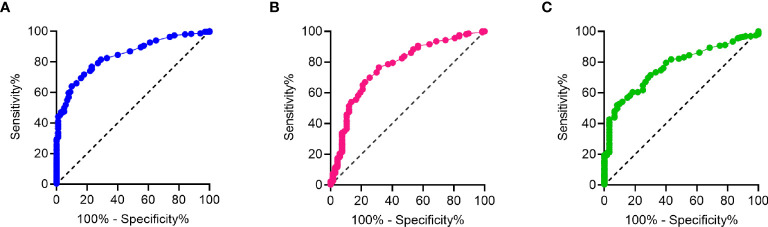
ROC analysis of CCl21 levels from ELISA 1, Luminex and ELISA 2. **(A)** ROC analysis of CCL21 levels from ELISA 1 with AUC=0.843, 95% CI = 0.084-0.883 and p<0.001, **(B)** ROC analysis of CCL21 levels from Luminex with AUC=0.772, 95% CI = 0.711-0.833 and p<0.001, and **(C)** ROC analysis of CCL21 levels from ELISA 2 with AUC=0.770, 95% CI = 0.711-0.830 and p<0.001.

In ELISA 1, a cut off value in which CCL21 was considered “high” or “low” was determined by ROC to be 0.29 (AUC=0.843, 95% CI = 0.084-0.883, p<0.001) (**Figure 5A**). Of the 41 patients with PAH, 4.9% had “low” levels of CCL21, while 95.1% had “high” levels of CCL21, p=0.037.

The cut off for “high” and “low” CCL21 in the Luminex was calculated to be 0.21 (AUC=0.772, 95% CI = 0.711-0.833, p<0.001) (**Figure 5B**). Of the 72 patients with PAH, 23.1% had “low” levels of CCL21, while the remaining 76.9% had “high” levels of CCL21, p=0.904.

Like ELISA 1, the cut off for “high” and “low” CCL21 value was 0.29 (AUC=0.770, 95% CI = 0.711-0.830, p<0.001) (**Figure 5C**). Of the 51 patients with PAH, 11.8% had “low” levels of CCL21, while the remaining 88.2% had “high” levels of CCL21, p=0.904.

### Association and prediction of CCL21 and PAH

In the previous study, ELISA 1, multivariable logistic- and cox-regression analysis showed that CCL21, anti-centromenre antibody (ACA), systolic pulmonary artery pressure (sPAP) and diffusing capacity of carbon monoxide (DLCO) were significantly associated with PAH and predictive of PAH (15). As shown in **Figure 3**, 26 patients form ELISA 1 were included in ELISA 2, as well as 25 other SSc-PAH patients. By performing multivariable logistic- and cox-regression analysis in the ELISA 2 cohort, we found that CCL21 were borderline significantly associated with PAH and sPAP were significantly associated with PAH (OR 4.53 (95% CI 0.98-20.92), p=0.053 and OR 1.06 (95% CI 1.00-1.11), p=0.037, respectively) and predictive for PAH (HR 1.58 (95% CI 1.02-2.44), p=0.038 and HR=1.03 (95% CI 1.02-1.05), p<0.001, respectively), shown in **Table 4**. ACA and DLCO however, were not significantly associated with PAH or predictive for PAH in multivariable logistic- and cox-regression analysis (**Table 3**).

**Table 3:** Multivariable logistic and cox regression analysis showing CCL21 and other variables associated with PAH and predictive of PAH.

Variable	Associated with PAH		Predictive of PAH	
	OR (95% CI)	p-value	HR (95% CI)	p-value
**CCL21, per SD**	4.53 (0.98-20.92)	0.053	1.58 (1.02-2.44)	0.038
**ACA**	2.86 (0.44-18.70)	0.274	0.91 (0.37-2.16)	0.823
**sPAP (mmHg)**	1.06 (1.00-1.11)	0.037	1.03 (1.02-1.05)	<0.001
**DLCO, %**	0.98 (0.93-1.05)	0.732	0.99 (0.97-1.01)	0.403

PAH, pulmonary arterial hypertension; OR, Odds ratio; CI, confidence interval; HR, Hazard ratio; SD, standard deviation; sPAP, systolic pulmonary artery pressure on echocardiography; DCLO, diffusing capacity of carbon monoxide.

### Serum anti-CCL21 antibodies in SSc and other rheumatic diseases

**Figure 6:**
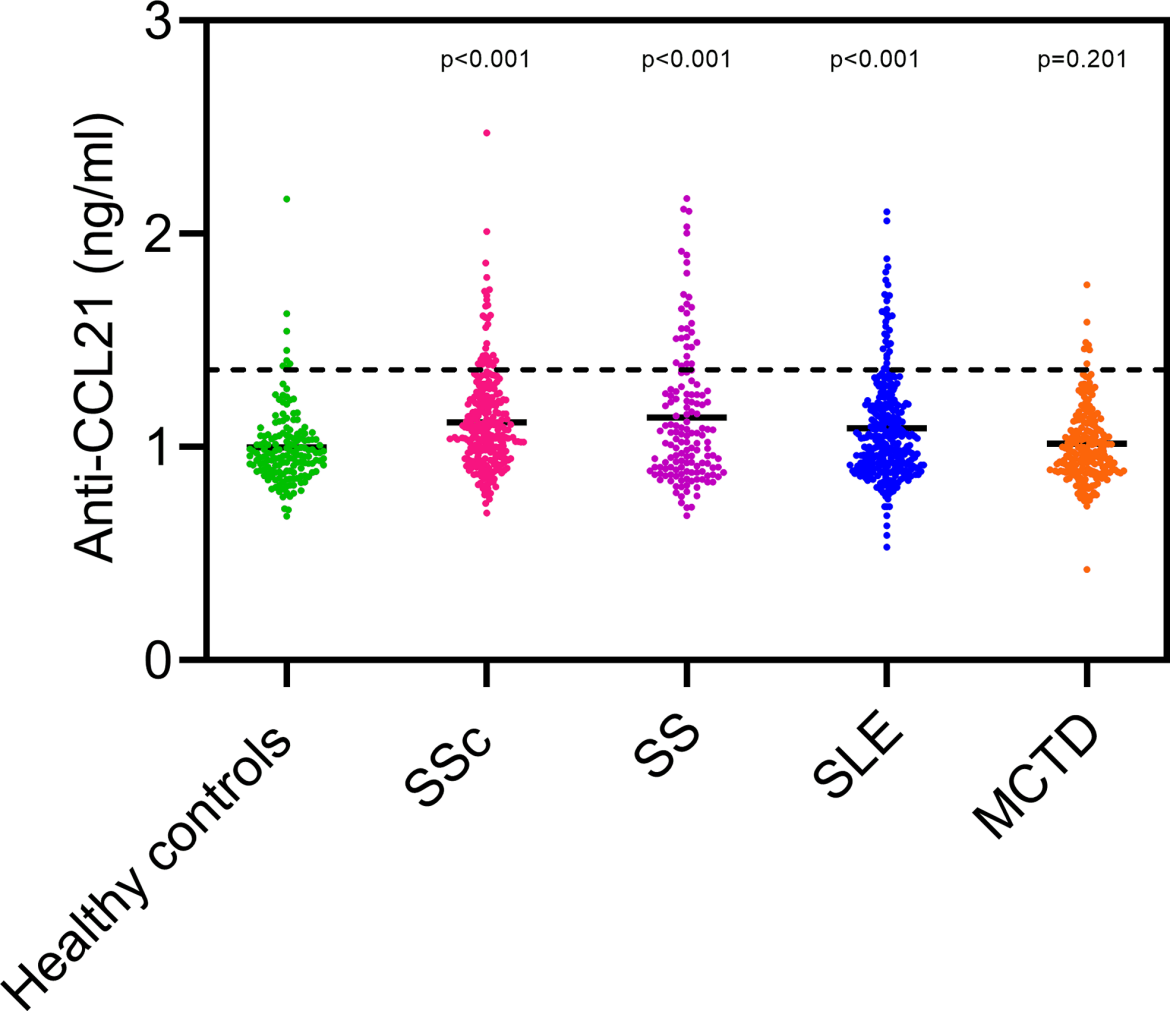
Serum anti-CCL21 antibody level in healthy controls and patients with different systemic connective tissue diseases. The dots in healthy controls (green), Systemic sclerosis (SSc; pink), primary Sjøgren syndrome (SS; purple), Systemic lupus erythematosus (SLE; blue) and Mixed connective tissue disease (MCTD; orange) shows the level of anti-CCL21 in the different patients. The dotted line represents the cut off value for considering the patients positive or negative for anti-CCL21, in which the patients above the dotted line are positive for anti-CCL21 antibodies. Levels of anti-CCL21 antibodies were significantly higher in patients with SSc, SS and SLE than in healthy controls (p-value).

Presence of circulating anti-CCL21 antibodies that specifically bind CCL21, and reduce availability of the chemokine for ELISA capture, could potentially explain finding of low CCL21 levels in some SSc patients with PAH. To explore this possibility, we searched for anti-CCL21 antibodies in SSc patients with and without PAH using an in-house multiplex bead assay. Additionally, to assess specificity, we tested for anti-CCL21 antibodies in healthy controls and cohorts of patients with other, well-characterized systemic connective tissue diseases. Levels of anti-CCL21 antibodies were significantly higher in patients with SSc, SS and SLE than in the healthy controls but not in MCTD. There was, however, no difference between anti-CCL21 levels in SSc and the other systemic rheumatic diseases (**Figure 6**).

Of the SSc patients, 17/300 (5.7%) had anti-CCL21 antibody levels above the cut-off value and were defined as anti-CCL21 antibody positive. Among these 17 patients, seven had conducted RHC. Of these seven, four (23.5%) were diagnosed with PAH, one (5.9%) had PH-ILD, and one (5.9%) had borderline mPAP, one had normal pulmonary pressure (5.9%). The remaining 10 (58.8%) had not conducted RHC.

**Figure 7:**
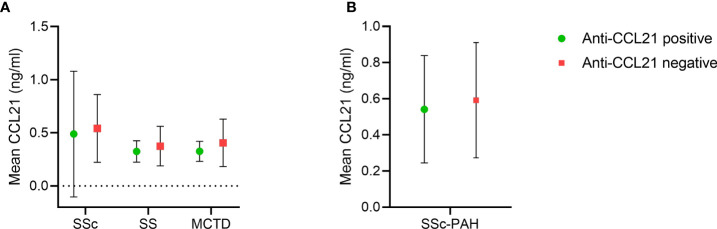
CCL21 levels in SSc, SS and MCTD of anti-CCL21 antibody positive and negative patients. **(A)** Mean serum levels of CCL21 in anti-CCL21 antibody positive (green) and anti-CCL21 antibody negative (red) patients in SSc, SS and MCTD. **(B)** Mean serum levels of CCL21 of anti-CCL21 antibody positive (green) and anti-CCL21 antibody negative (red) patients in SSc patients diagnosed with PAH.

We had available data on CCL21 levels by ELISA in 216 of the SSc serum samples analyzed for anti-CCL21 antibodies, and from ELISA1, where SS and MCTD were included as disease controls. These samples included paired data on CCL21 serum levels and anti-CCl21 antibodies in 85 SS patients and 143 MCTD patients. We used these data to compare CCL21 levels in patients positive and negative for anti-CCL21 antibodies. We found that the mean levels of CCL21 in anti-CCL21 antibody positive patients were 0.49 ± 0.59 ng/ml, 0.33 ± 0.10 ng/ml and 0.33 ± 0.09 ng/ml for SSc, SS and MCTD respectively. For the anti-CCL21 antibody negative patients, the mean levels of CCL21 were 0.46 ± 0.53 ng/ml, 0.38 ± 0.19 ng/ml and 0.41 ± 0.22 ng/ml for SSc, SS and MCTD, respectively (**Figure 7A**). Within the patient groups, there were no significant difference in the CCL21 levels between those who were anti-CCL21 antibody positive or negative (SSc: p=0.84, SS: p=0.88, MCTD: p=0.92). In the subset of SSc patients with PAH verified by RHC, four were anti-CCL21 antibody positive while 23 were anti-CCL21 antibody negative. The mean levels of CCL21 were 0.54 ± 0.28 ng/ml in the anti-CCL21 positive patients and 0.59 ± 0.32 ng/ml in the negative patients, with no significant difference between the groups (p=0.77) (**Figure 7B**).

### Immunoprecipitation, western blot and mass spectrometry

**Figure 8:**
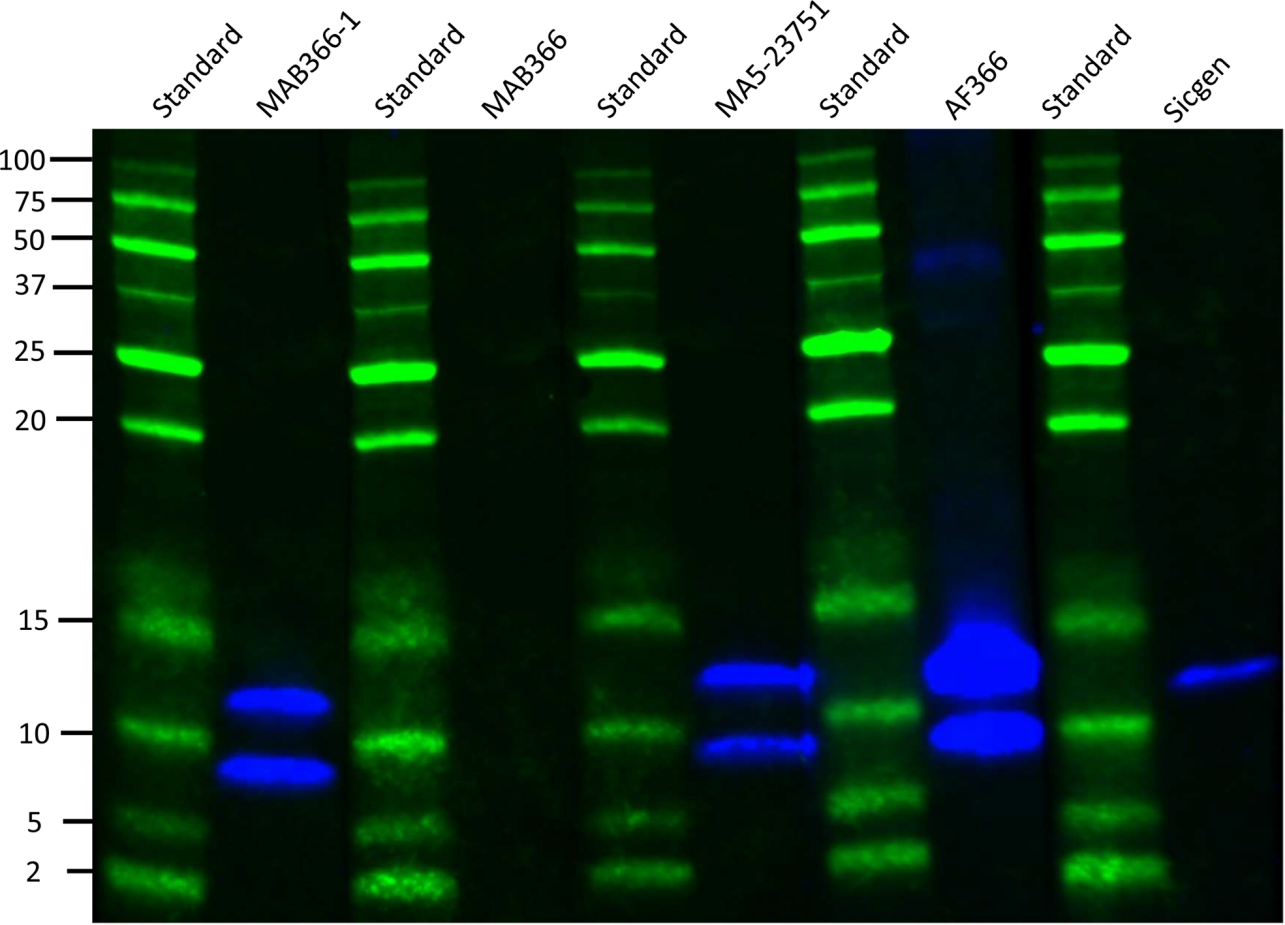
Test of CCL21-antibodies using Western blot analysis. The Western blot analysis showed that three of the CCL21 antibodies detected both full length and tailless CCL21 (four left lanes). The custom-made anti-C-terminal tail of CCL21 antibody from Sicgen only detected full length CCL21 (right lane).

By SDS-PAGE analysis and Western blot, we found that three out of four commercial anti CCL21 monoclonal antibodies detected both the full length recombinant CCL21 protein and the recombinant CCL21 protein lacking the C-terminal, defined as tailless CCL21. Additionally, and as expected, we found that the custom-made anti-C-terminal CCL21 antibody from Sicgen detected the full length CCL21 protein, but not tailless CCL21 (**Figure 8**).

**Figure 9:**
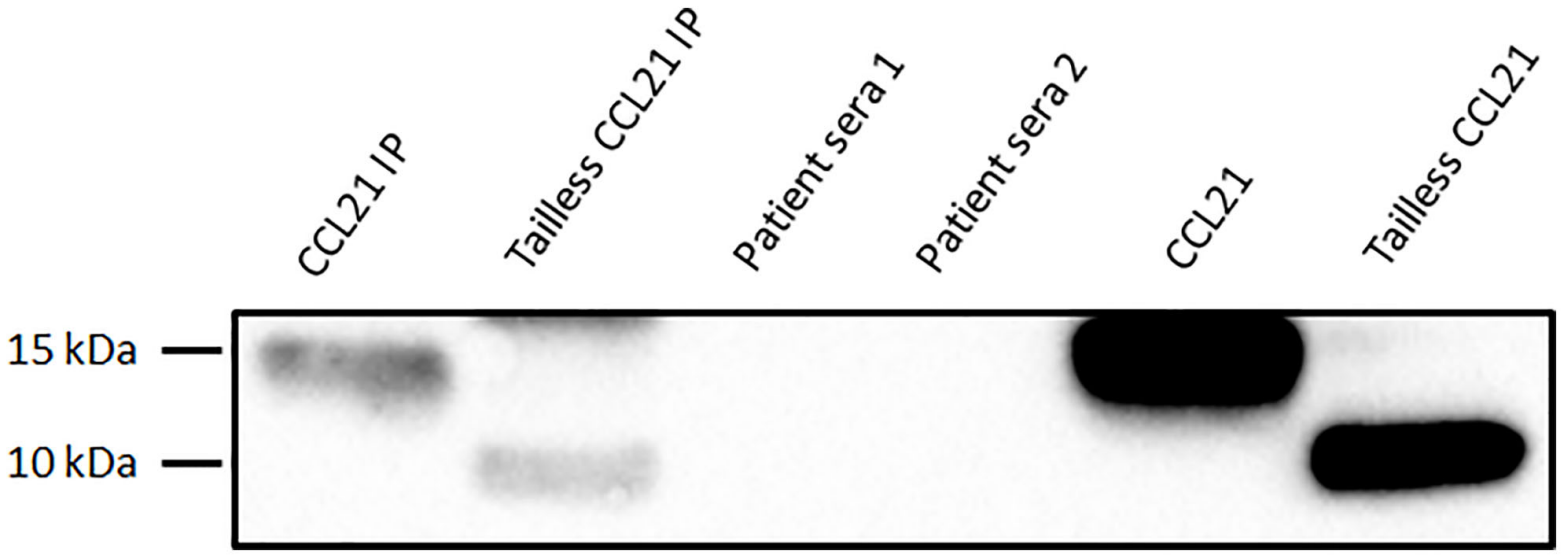
Western blot of SSc serum samples. The four first lanes show the IP product of 1: costume made CCL21, 2: Tailless CCL21, 3: Patient serum 1, and 4: patient serum 2. The last two lanes CCL21 and Tailless CCL21 directly loaded on the gel.

For the further IP and MS experiments, we applied the anti-CCl21 mAb from ThermoFisher (MA5-23751) and the anti-C-terminal tail of CCL21 antibody from Sicgen. Serum samples from two SSc patients displaying elevated levels of CCL21 (0.63 ng/ml and 2.05 ng/ml on ELISA 1) were analyzed by Western blot to determine if both full length CCL21 and tailless CCL21 were present in the serum. Custom made CCL21 (14,656 kDa) and Tailless CCL21 (8.85 kDa) were used as positive controls. In the two first lanes of the blot, we show custom made full length and tailless CCL21 which have been through the same IP process as serum samples from patients in lane 3 and 4. In the two last lanes we show custom made full length and tailless CCL21 before IP (**Figure 9**). When comparing CCL21 IP and tailless CCL21 IP to CCL21 and tailless CCL21 loaded directly on the gel, the volume detected on the gel are 17-fold higher for CCL21 and 19-fold higher for tailless CCL21 directly loaded. Unfortunately, we were not able to detect CCL21 in any of the two SSc serum samples applied on the gel.

Given the negative western blot, we proceeded with MS. Before MS the IP product of serum samples from the two SSc patients, we tested both the anti-CCl21 mAb from ThermoFisher (MA5-23751) and the anti-C-terminal tail of CCL21 antibody from Sicgen on custom made CCL21 in MS analysis. Unfortunately, only the ThermoFisher antibody detected enough peptides for use in MS of the IP product. By MS of the IP product, we were able to detect a number of peptides located within amino acid (aa) 23-102 of CCL21, shown in **Table 4**. No peptides from the C-terminal (103-134 aa) of CCL21 were detected.

**Table 4:** Peptides detected by MS of CCL21 immunoprecipitated from SSc patient serum.

Sequence	Length (aa)	Mass (kDA)	Location in CCL21 (1-134 aa)
**ELWVQQLMQHLDKTPSPQKPAQG**	23	2658,3588	80-102
**SDGGAQDCCLK**	11	1209,4754	23-34
**TPSPQKPAQG**	10	1009,5193	93-102
**ELWVQQLMQHLDK**	13	1666,8501	80-92
**KQEPSLGCSIPAILFLPR**	18	2025,1081	50-67
**KRSQAELCADPK**	12	1401,7034	68-79
**QEPSLGCSIPAILFLPR**	17	1897,0132	51-67
**SQAELCADPK**	10	1117,5074	70-79

aa, amino acids; kDA, kilo Dalton.

## Discussion

PAH remains a major clinical challenge in SSc. PAH detection algorithms have shown efficacy in reducing diagnostic delay, but they are costly, time-consuming and necessary to perform across the whole SSc population. Therefore, we are still in need of novel biomarkers to aid risk stratification of patients for screening algorithms, earlier diagnosis and targeted interventions. We previously identified circulating CCL21 as a potential predictor of PAH in SSc. Moving on, we in approached several important questions related to relevance, applicability and biology of CCL21 in SSc-PAH. Major findings were; (i) CCL21 expression was mainly on airway epithelium in lung tissues from SSc patients with PH. (ii) Extension and replication of circulating CCL21 as a biomarker was promising with ELISA assay in two new and independent cohorts. (iii) Inability, to replicate ELISA results on the more versatile Luminex platform. (iv) Anti-CCL21 auto-antibodies were present in multiple CTDs, but they did not seem to be of major importance in SSc-PAH. (v) MS data indicated predominance of truncated CCL21, rather than full-length protein in blood from SSc-PAH patients.

In the IHC stanings of lung tissue from SSc patients diagnosed with PAH, CCL21 were primarily expressed in the airway epithelial cells (AECs). AECs are known to contribute to the pathogenesis of airway disease (47). Following direct physical injury or exposure to environmental stress, normal epithelial cells release a number of cytokines and chemokines (48). A previous study has reported that AECs collected from asthmatic lungs have immediate cytokine and chemokine response to injury compared to non-asthmatic, normal AEC (49). It has also been shown that AEC are involved in pulmonary vascular remodeling (50) and that continuous injury to AEC might be related to PAH (51). A study has also shown that AEC conditioning of DC induced the upregulation of genes that mediate important DC functions (52). As mentioned in the introductions, CCL21 is important for orienting naïve T cells to DC in lymphatic vessel (32).

Additionally, we detected CCL21 expression in alveolar macrophages. Macrophages are innate immune cells specialized to maintain tissue homeostasis, and they rapidly change phenotype when inflammation ensue, as shown for alveolar macrophages in lung tissue (53, 54). While macrophages appear important in the development of PAH, the precise roles of these cells in the pathogenesis of SSc-PAH remains unknown (55). Notably, a study in rheumatoid arthritis shows that CCL21/CCR7 signaling increases the number of macrophages in synovial tissue and promotes joint inflammation, making CCL21 an attractive target for therapy. In the synovial tissue, the CCL21 induced macrophages secret IL-6 and IL-23, driving differentiation of naïve T cells to IL-17 producing T helper cells 17 (Th17) (56). This finding is of general interest since Th17 cells appear to play important roles in many autoimmune diseases (57, 58). In SSc, several studies indicate key roles of Th17 cells in disease pathogenesis, but the mechanism by which these cells operate have still not been fully worked out (59–61). Regarding SSc-PAH, data are limited, but there is one study indicating Th17 polarization by flow cytometry of lymphocytes from PAH patients (62). While signaling through CCL21-CCR7 axis occurs in numerous cell types, and has been shown to modulate a number of biological processes, it is not clear which of these are most relevant for SSc-PAH. Immune cells, such as the Th17 cells discussed above, are obvious candidates, but so are also endothelial cell in vascular and lymphatics. Regarding potential mechanisms for vascular endothelial cell involvement in SSc-PAH, it is interesting to note that studies indicate links between CCL21, tumor necrosis factor (TNF)-α and vascular endothelial growth factor in different mouse models (63–67). The links and interaction of CCL21 and other cytokines playing a role in SSc-PAH will be interesting for further investigation.

In this study, a major aim was to extend previous results indicating CCL21 as a circulating biomarker for SSc-PAH. For this purpose we assessed serum CCL21 in two large, well-characterized SSc cohorts by Luminex xMAP analysis, a high throughput, and timesaving method for the measurement of chemokines and biomarker in serum samples (33). Unexpectedly, we found that correlation between serum levels of CCL21 determined by our previous ELISA (ELISA 1) and the Luminex xMAP assay were weak, showing poor comparability between these two methods. To further clarify this issue, we set up a new, large ELISA (ELISA 2) analysis with serum samples included in both ELISA 1 and Luminex xMAP. ELISA 2 and Luminex xMAP did also have week correlation between the levels of CCL21, again showing poor comparability between these methods. The correlation of CCL21 levels in ELISA 1 and ELISA 2, however, were strong and showed high reproducibility. Moreover, and importantly, by ELISA2, we again demonstrate CCL21 association to SSc-PAH and its predictive value of PAH development in new independent cohorts, indicating robustness of these findings. Together, this circulating CCL21 analyses indicate that ELISA and Luminex are not comparable methods for CCL21 in SSc, and support the notion that ELISA may be more sensitive in the concentration range of CCL21 than the multiplex assay and associated with PAH and predicts PAH in SSc.

Recent studies indicate important roles in immune-mediated disorders of auto-antibodies targeting immune-related molecules, such as cytokines and chemokines. Here, we addressed the possibility that anti-CCL21 antibodies could be involved in SSc. We found that anti-CCL21 antibodies were not specific for SSc compared to other rheumatic diseases, and was not associated with PAH in SSc patients. Patients who were positive for anti-CCL21 antibodies had varying levels of CCL21 ranging from 0.09 ng/ml to 2.45 ng/ml. Of these SSc patients, four had PAH. The role of antibodies should be addressed in a larger cohort of SSc patients developing PAH over time.

In the SDS-PAGE/western blot, where CCL21- and tailless CCL21 IP products were loaded on a gel together with CCL21 and tailless CCL21 loaded directly on the gel we saw less signal for the IP product compared to directly loading. The concentration of CCL21 and tailless CCL21 loaded directly on the gel were in the same range as the starting concentration of CCL21 and tailless CCL21 before IP. This shows that a major part of the product is lost during the IP process. The concentration of CCL21 in SSc serum samples were much lower than the starting concentration of purchased CCL21 and tailless CCL21 before IP. The observation that a large amount of the CCL21 product was lost during IP could explain why we were not able to detect CCL21 in the serum samples. In a search for western blot analyses of CCL21 in serum, we did not find any previous data. However, we found on the human protein atlas webpage (https://www.proteinatlas.org/ENSG00000137077-CCL21/antibody) western blot analysis of CCL21 in plasma and other biological materials. CCL21 was detected in cell lines, liver and tonsil, but again not in plasma, underlying this most likely explanation.

Before MS analysis of the CCL21 IP product from SSc serum samples, we tested one antibody binding to an aa-sequence present in both full length CCL21 and tailless CCL21, and an antibody binding only to the C-terminal of CCL21 in IP analysis of full length CCL21. Unfortunately, the antibody detecting only the C-terminal of CCL21 did not bind enough CCL21 to proceed with this antibody for IP of SSc serum samples which contain a much lower concentration of CCL21. Therefore, we used the antibody binding both full length and tailless CCL21 for IP of SSc serum samples prior to MS. Even though we did not detect CCL21 from serum samples in western blot, peptides from CCL21 were detected by MS. Interestingly, while we identified multiple CCL21 peptides in the IP-product by MS, there were no peptides from the C-terminal of CCL21 by MS. This indicates truncated, tailless CCL21, and not full-length protein as the major circulating form in our patients.

A major strength of this study is the large cohort with serum samples from two independent cohorts used to investigate the level of CCL21 in SSc patients with comprehensive clinical data available. Another strength is that replicated CCL21 levels in serum samples from patients using ELISA in to different runs with some overlapping patients and its potential to associate and predict with PAH. We also included large cohorts of patients with other CTDs (SLE, SS and MCTD) and healthy controls for comparison.

One weakness of this study is the low number patients with lung tissue samples for IHC. As mentioned, collection of lung biopsies for diagnostic or therapeutic purpose in patients with high pulmonary arterial pressure is not recommended. The fact that we did not manage to reproduce the CCL21 results from ELISA using the less time consuming and high throughput analysis, Luminex is a weakness for clinical application of CCL21 in clinical practice in the future.

In conclusion, this study demonstrates expression of CCL21 in epithelial lung tissue from SSc patients developing PAH, and indicates that CCL21 in SSc circulates as a truncated protein. We extend previous observations indicating biomarker potential of CCL21 both associated with PAH and its predictive ability of PAH development, but find that Luminex is not suitable as platform for assessment of CCL21 as a circulating biomarker. Finally, *in vivo* generated anti-CCL21 antibodies exists in SSc, but do not appear to modify serum CCL21 levels in patients with SSc-PAH.

## Data availability statement

The raw data supporting the conclusions of this article will be made available by the authors, without undue reservation.

## Ethics statement

The studies involving human participants were reviewed and approved by Data Protection Authority in Norway (No.2006/119), BASEC KEK-ZH in Zurich (No.2018-01873) and BASEC in Zurich (No.PB_2016-020114). The patients/participants provided their written informed consent to participate in this study.

## Author contributions

Substantial contributions to study conception and design: HD, A-MH-V, ØMo, JB. Substantial contributions to acquisition of data: All authors. Substantial contributions to analysis and interpretation of data: HD, A-MH-V, ØMo, JB, AM, OD, SJ, VP. Drafting the article for important intellectual content: All authors. All authors contributed to the article and approved the submitted version.

## Funding

This study was financed by a grant from Helse Sør-Øst (project number: 2016001). The award number for the grant with Cedar-Sinai is P01HL108793.

## Conflict of interest

HF has received personal fees from Bayer and non-financial support <$10 000 from GSK and Actelion, outside the submitted work. ØMi has received honoraria from Boehringer Ingelheim and Jannsen. AA has a consultancy relationship with and has received travel support and honoraria from Jannsen. OD has a consultancy relationship with and/or has received research funding from and/or has served as a speaker for the following companies in the area of potential treatments for systemic sclerosis and its complications in the last three calendar years: Abbvie, Acceleron, Alcimed, Amgen, AnaMar, Arxx, AstraZeneca, Baecon, Blade, Bayer, Boehringer Ingelheim, Corbus, CSL Behring, 4P Science, Galapagos, Glenmark, Horizon, Inventiva, Janssen, Kymera, Lupin, Medscape, Miltenyi Biotec, Mitsubishi Tanabe, MSD, Novartis, Prometheus, Redxpharma, Roivant, Sanofi and Topadur. OD also has a patent issued “mir-29 for the treatment of systemic sclerosis” (US8247389, EP2331143). JB has received honoraria from Boehringer Ingelheim. A-MH-V has received grants, personal fees and non-financial support >$10 000 from Boehringer Ingelheim, and personal fees <$10 000 from Actelion, ARXX, Bayer, Jansen, Medscape, MSD and Roche outside the submitted work.

The remaining authors declare that the research was conducted in the absence of any commercial or financial relationships that could be construed as a potential conflict of interest.

## Publisher’s note

All claims expressed in this article are solely those of the authors and do not necessarily represent those of their affiliated organizations, or those of the publisher, the editors and the reviewers. Any product that may be evaluated in this article, or claim that may be made by its manufacturer, is not guaranteed or endorsed by the publisher.
